# Phase II placebo-controlled study of nepafenac ophthalmic suspension 0.1% for postoperative inflammation and ocular pain associated with cataract surgery in Japanese patients

**DOI:** 10.1007/s12348-011-0036-8

**Published:** 2011-09-20

**Authors:** Jiro Numaga

**Affiliations:** Department of Ophthalmology, Tokyo Metropolitan Geriatric Hospital, 35-2 Sakae-cho, Itabashi-ku, Tokyo 173-0015 Japan

**Keywords:** Japanese, Nepafenac, Placebo control, Postoperative inflammation, Ocular pain

## Abstract

**Objective:**

This study aimed to examine the efficacy and safety of nepafenac ophthalmic suspension compared to placebo in the management of postoperative inflammation and ocular pain in Japanese patients undergoing cataract surgery.

**Methods:**

This was a multicenter, randomized, double-masked, placebo-controlled clinical study. Patients received nepafenac or placebo TID beginning 1 day before cataract surgery and continuing on the day of surgery for 14 days. One additional drop was administered on the day of surgery. The primary efficacy variables were the percentage of patients cured at postoperative day 14 visit (cure defined as aqueous cells score + aqueous flare score = 0) and the percentage of patients who were pain free at all postoperative visits.

**Results:**

The cure rate on day 14 after surgery was 71.4% (75/105) in the nepafenac group and 28.6% (30/105) in the placebo group, showing a significant difference in cure rate between groups. The nepafenac group demonstrated higher cure rates than those in the placebo group, with a significant difference in cure rate on days 7 and 14 postoperatively. The ocular pain-free rate was 96.2% (102/106) in the nepafenac group and 67.6% (71/105) in the placebo group, showing a significant difference between groups. Concerning adverse events (AEs), 26 AEs were reported in 21 subjects (19.6%) in the nepafenac group and 31 AEs were reported in 24 subjects (22.4%) in the placebo group.

**Conclusion:**

Nepafenac ophthalmic suspension is a nonsteroidal anti-inflammatory drug effective in the prevention of postoperative inflammation and ocular pain associated with cataract surgery.

## Introduction

Nepafenac is a nonsteroidal anti-inflammatory drug (NSAID) and a prodrug that is metabolized into its active form, amfenac, following ocular administration. Delivering amfenac as a prodrug permits increased corneal penetration and high aqueous concentration of the compound [1]. Amfenac suppresses prostaglandin production by inhibiting cyclooxygenase (COX), as do other NSAIDs [1]. Following the administration of diclofenac, total prostaglandin production by the rabbit iris–ciliary body was inhibited by approximately 40% within 5 min and remained unchanged thereafter, while nepafenac exhibited an inhibitory effect on prostaglandin production between 5 and 10 min after administration, with the inhibitory rate exceeding 90% between 40 and 80 min after administration [2]. The inhibitory effect of nepafenac on extravascular protein exudation into the aqueous humor of the rabbit continued for 8 h after administration while diclofenac inhibited vascular permeation up to 4 h after administration but exhibited no significant inhibitory effect 8 h after administration [2]. Since its launch in the USA in 2005, nepafenac 0.1% has been widely used in clinical practice in more than 70 countries worldwide.

To receive marketing approval for the drug in Japan, we conducted a placebo-controlled, multicenter trial to evaluate the efficacy and safety of the drug in the management of postoperative inflammation and ocular pain in Japanese patients undergoing cataract surgery with phacoemulsification and aspiration (PEA).

## Materials and methods

### Study drug

**Fig. 1:**
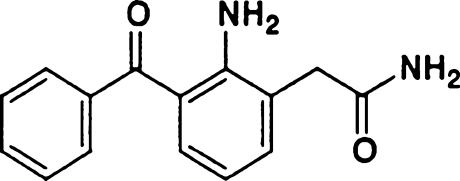
Chemical structure of nepafenac

Nepafenac ophthalmic suspension containing 1 mg of nepafenac (Fig. 1) per 1 ml and placebo with the same formulation as nepafenac suspension excluding nepafenac ingredient supplied in identical, opaque 5-ml bottles.

### Study design

This was a multicenter, randomized, double-masked, placebo-controlled study conducted at 11 Japanese sites (Table 1) from August 2006 to December 2006. This study was conducted in accordance with the Declaration of Helsinki with approval from the institutional review boards. Patients were randomly assigned to receive nepafenac or placebo. Patients instilled one drop in the operative eye three times a day. They began dosing 1 day before surgery and continued on the day of surgery and for 14 days thereafter. On the day of surgery, an additional dose was administered before surgery. The use of other NSAIDs and steroids were prohibited. The test drugs are blinded to the investigators and subjects except for the appointed controller of study drugs.

**Table 1:** Investigators and institutions

No.	Institutions	Investigators
1	Ohtsuka Eye Hospital	Makoto Higuchi
2	Mitani Eye Clinic	Kiichiro Mitani
3	Muramatsu Eye Clinic	Tomoyuki Muramatsu
4	Nakajima Eye Clinic	Toru Nakajima
5	Kikukawa Eye Clinic	Koichiro Ikeda
6	Tokai Eye Clinic	Yoshihide Nakai
7	Kitano Hospital	Isao Saito
8	Nishi Eye Hospital	Hiroki Iwanishi
9	Hirota Eye Clinic	Atsushi Hirota
10	Ohshima Hospital of Ophthalmology	Nobuyuki Yabe
11	Hayashi Eye Hospital	Ken Hayashi

### Subjects

Patients 20 years of age or older scheduled to undergo cataract extraction by PEA and implantation of an intraocular lens (IOL) were eligible for the study. Each patient was given sufficient explanation about the purpose and details of the study and provided written consent to participate in the study. Patients were excluded from enrollment if they used topical ocular or systemic steroids within 14 days of surgery or used topical ocular or systemic NSAIDs within 7 days of surgery, except an allowed daily dose of low-dose aspirin. Patients were also excluded if they planned to have cataract surgery in their fellow, non-study eye before the 14-day postoperative study visit or had any cells, flare, or ocular pain in the operative eye at the screening examination, had planned multiple surgical procedures, exfoliation syndrome, ocular trauma, inflammatory eye disease, diabetic retinopathy which needed medical treatment, and uncontrolled diabetes mellitus, or had grade 3 or higher cataracts according to Emery–Little classification [3].

### Examination items and schedule

The examination schedule is shown in Table 2. Best-corrected visual acuity (BCVA) and intraocular pressure (IOP) measurement, ocular pain assessment, slit lamp examination, and laser flare cell meter (LFCM) measurement were performed before and 1, 3, 7, and 14 days after surgery. A fundus examination was performed before and 7 and 14 days after surgery. Blood pressure/pulse rate measurement was performed before and at the day of surgery and 1, 3, 7, and 14 days after surgery. A laboratory examination was performed before and 14 days after surgery.

**Table 2:**
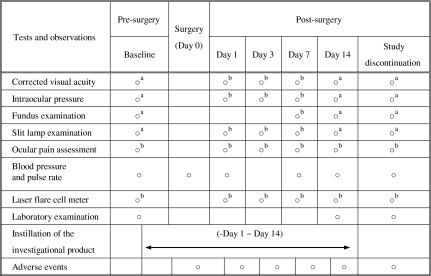
The examination schedule

^a^Both eyes

^b^Study (operative) eye

### Efficacy evaluation

The grading criteria and scale for clinical symptom and signs are summarized in Table 3. The criteria and scale are the same as previous analysis of nepafenac [4–6]. Patients who received the test drug, completed surgery, and had at least one follow-up visit were included in the intention-to-treat (ITT) analyses. Primary efficacy analyses were conducted on the ITT data set. The primary efficacy variables were the cure rate (the percentage of subjects with an aqueous cell score + aqueous flare score = 0) at day 14 after surgery and the ocular pain-free rate (the percentage of subjects with an ocular pain score of 0) at all postoperative visits. Also, the cure rate and the ocular pain-free rate at each postoperative visit were compared between two groups. The secondary variables were aqueous cell score, aqueous flare score, cell count, flare value, and the treatment failure rate (the percentage of subjects with either an aqueous cell score or an aqueous flare score of 3 or more, or an ocular pain score of 4 or more) at each postoperative visit. Cell count and flare value were measured with LFCM.

**Table 3:** Grading criteria for clinical symptom and signs

	Grading criteria
Ocular pain	0: None—absence of pain
	1: Trace—slight sensation of pain or discomfort
	2: Mild—mild, tolerable aching of the eye
	3: Moderate—moderate and prolonged aching sufficient to require the use of analgesics
	4: Moderately severe—prolonged intense aching requiring the use of analgesics
	5: Severe—prolonged sharp ocular or periocular pain
Flare	Use a 1 mm wide beam of the slit lamp aimed at the center of the pupil
	0: None—no visible flare when compared with the normal eye
	1: Mild—flare visible against dark pupillary background but not visible against iris background
	2: Moderate—flare is visible with the slit lamp beam aimed onto the iris surface as well as the dark pupillary background
	3: Severe very dense flare. May also present as a “hazy” appearance of anterior segment structures when viewed with low power magnification of the slit lamp. Presents as a pronounced Tyndall effect
Cell	Use narrow slit beam (0.5 mm in width and at least 8 mm in length) at maximum luminance. Pigment and red blood cells are to be ignored.
	0: None
	1: 1 to 5 cells
	2: 6 to 15 cells
	3: 16 to 30 cells
	4: Greater than 30 cells

### Safety evaluation

All patients receiving the test drug were included in the safety analysis. Safety evaluation included incidence of adverse events (AEs), blood pressure/pulse rate, laboratory tests, BCVA, IOP, slit lamp examination (bulbar conjunctival injection, corneal edema, and chemosis), and fundus examination (retina, macula lutea, choroid, and optic nerve).

### Statistical analysis

For the primary variables of cure rate at day 14 and the ocular pain-free rate at all postoperative visits, a Chi-square test of independence was conducted to assess the superiority of nepafenac relative to placebo. Multiplicity adjustment for the primary endpoints was made by sequential testing with fixed sequences, in which the cure rate was analyzed first followed by the ocular pain-free rate. And a Chi-square test or a Fisher’s exact test was performed for the cure rate and the ocular pain-free rate at each study visit.

The analysis of aqueous cells score, flare scores, cell counts, and flare value was conducted with a repeated measures analysis of variance at postoperative visits. A two-sample *t* test was used for the comparisons in aqueous cells score and flare score at baseline between two groups. The comparisons between nepafenac and placebo in treatment failures at each postoperative visit were made using nonlinear mixed model with maximum likelihood estimation from NLMixed procedure of SAS software. Demographics data were compared between two groups using by a Chi-square test. A *P* value less than 0.05 indicated statistical significance. Data analyses were performed using SAS software, version 9.1.

## Results

### Patient population and demographics

Two hundred fifteen patients, 108 treated with nepafenac and 107 treated with placebo, were enrolled in this study. One patient discontinued prior to the use of the study medication for patient decision. Two hundred fourteen remaining patients received at least one dose of test drug and were therefore included in the safety analysis. Three of 214 patients discontinued from the study prior to or at the time of surgery for patient decision (*n* = 1), the use of NSAIDs (*n* = 1) and investigator decision due to surgical difficulties. These three patients were excluded in the ITT population. A total of 211 patients, 106 treated with nepafenac and 105 treated with placebo were included in the ITT population.

The demographics data for the patients included in the ITT data set are summarized in Table 4, and the demographics data by institution are showed in Table 5. As indicated in Table 4, there were no statistically significant differences in gender, age, and Emery’s classification between treatment groups.

**Table 4:** Demographic data (ITT)

	Total		Nepafenac group		Placebo group		*P* value^a^
	*N*	%	*N*	%	*N*	%	
Total	211	100.0	106	50.2	105	49.8	
Gender							
Male	98	46.4	49	46.2	49	46.7	0.9489
Female	113	53.6	57	53.8	56	53.3	
Age							
18 to 64 years	59	28.0	24	22.6	35	33.3	0.0836
≥ 65 years	152	72.0	82	77.4	70	66.7	
Emery’s classification							
1	50	23.7	28	26.4	22	21.0	0.3508
2	161	76.3	78	73.6	83	79.0	

^a^From Chi-square

**Table 5:** Demographic data by institution (ITT)

			Gender				Age				Emery’s classification			
			Male		Female		<65		≥65		1		2	
			*N*	%	*N*	%	*N*	%	*N*	%	*N*	%	*N*	%
Total		Nepafenac	49	46.2	57	53.8	24	22.6	82	77.4	28	26.4	78	73.6
		Placebo	49	46.7	56	53.3	35	33.3	70	66.7	22	21.0	83	79.0
Investigator no.	1	Nepafenac	4	66.7	2	33.3	2	33.3	4	66.7	4	66.7	2	33.3
		Placebo	2	33.3	4	66.7	3	50.0	3	50.0	5	83.3	1	16.7
	2	Nepafenac	5	41.7	7	58.3	3	25.0	9	75.0	5	41.7	7	58.3
		Placebo	6	50.0	6	50.0	1	8.3	11	91.7	5	41.7	7	58.3
	3	Nepafenac	5	33.3	10	66.7	2	13.3	13	86.7	0	0.0	15	100.0
		Placebo	6	40.0	9	60.0	2	13.3	13	86.7	0	0.0	15	100.0
	4	Nepafenac	6	75.0	2	25.0	0	0.0	8	100.0	6	75.0	2	25.0
		Placebo	4	50.0	4	50.0	2	25.0	6	75.0	5	62.5	3	37.5
	5	Nepafenac	7	43.8	9	56.3	2	12.5	14	87.5	0	0.0	16	100.0
		Placebo	7	43.8	9	56.3	5	31.3	11	68.8	0	0.0	16	100.0
	6	Nepafenac	3	60.0	2	40.0	1	20.0	4	80.0	0	0.0	5	100.0
		Placebo	2	33.3	4	66.7	3	50.0	3	50.0	0	0.0	6	100.0
	7	Nepafenac	2	22.2	7	77.8	2	22.2	7	77.8	2	22.2	7	77.8
		Placebo	3	50.0	3	50.0	2	33.3	4	66.7	2	33.3	4	66.7
	8	Nepafenac	3	37.5	5	62.5	3	37.5	5	62.5	2	25.0	6	75.0
		Placebo	4	44.4	5	55.6	4	44.4	5	55.6	1	11.1	8	88.9
	9	Nepafenac	5	55.6	4	44.4	2	22.2	7	77.8	2	22.2	7	77.8
		Placebo	5	55.6	4	44.4	5	55.6	4	44.4	2	22.2	7	77.8
	10	Nepafenac	4	33.3	8	66.7	3	25.0	9	75.0	6	50.0	6	50.0
		Placebo	6	50.0	6	50.0	5	41.7	7	58.3	1	8.3	11	91.7
	11	Nepafenac	5	83.3	1	16.7	4	66.7	2	33.3	1	16.7	5	83.3
		Placebo	4	66.7	2	33.3	3	50.0	3	50.0	1	16.7	5	83.3

### Efficacy

There was a significant difference in cure rate at day 14 between nepafenac (71.4%, 75/105) and placebo (28.6%, 30/105) (*P* < 0.0001), demonstrating the effectiveness of nepafenac in reducing postoperative inflammation (Table 6). When comparing cure rates at each visit, nepafenac achieved significantly higher cure rates than placebo at days 7 and 14 (*P* < 0.0001 for days 7 and 14) (Table 6). The ocular pain-free rate was 96.2% (102/106) in the nepafenac group and 67.6% (71/105) in the placebo group, showing a significant difference between groups (*P* < 0.0001) (Table 7). The nepafenac group demonstrated significantly higher ocular pain-free rates than the placebo group at all postoperative visits (*P* = 0.0051 for day 1, *P* = 0.0003 for day 3, *P* = 0.0002 for day 7, and *P* = 0.0051 for day 14) (Table 8). In the nepafenac group, cell scores and flare scores measured at each postoperative visit decreased as time elapsed after surgery, showing the suppressive effect of nepafenac on postoperative inflammation. In contrast, no marked decrease was observed in either the cell or flare scores in the placebo group. Significant intergroup differences were observed in the cell score on day 7 and subsequent visits (*P* < 0.0001 for days 7 and 14) and in the flare score on day 3 and subsequent visits (*P* = 0.0041 for day 3 and *P* < 0.0001 for days 7 and 14) (Tables 9 and 10). Postoperative cell counts and flare values were measured with LFCM. Decreases in both cell counts and flare values were observed on day 3 and subsequent visits in the nepafenac group, whereas increases in both variables were observed on day 3 in the placebo group. Significant intergroup differences were observed in cell counts and flare values on day 3 and subsequent visits (Tables 11 and 12).

**Table 6:** Cure rate at each study visit (ITT)

Treatment	All^a^ (*N*)	Day 1		Day 3		Day 7		Day 14	
		*N*	%	*N*	%	*N*	%	*N*	%
Nepafenac group	105	2	1.9	22	21.0	53	50.5	75	71.4
Placebo group	105	2	1.9	16	15.2	20	19.0	30	28.6
*P* value			1.00		0.2822		<0.0001		<0.0001

Test = Chi-square (Fisher’s exact test if *N* < 5)

^a^One patient missing cure data

**Table 7:** Percent ocular pain free (ITT)

Treatment	Total	Pain free	
	*N*	*N*	%
Nepafenac group	106	102	96.2
Placebo group	105	71	67.6

Test = Chi-square, *P* < 0.0001

**Table 8:** Percentage of patients with no pain at each study visit (ITT)

Treatment	All (*N*)	Day 1		Day 3		Day 7		Day 14	
		*N*	%	*N*	%	*N*	%	*N*	%
Nepafenac group	106	103	97.2	104	98.1	103	97.2	103	97.2
Placebo group	105	91	86.7	88	83.8	85	81.0	91	86.7
*P* value			0.0051		0.0003		0.0002		0.0051

Test = Chi-square

**Table 9:** Aqueous cells score by treatment and visit (ITT)

		Baseline	Day 1	Day 3	Day 7	Day 14
Nepafenac group	Mean	0.0	1.3	0.9	0.5	0.3
	Std	0.0	0.6	0.7	0.6	0.6
	*N*	106	105	105	105	105
	Min	0	0	0	0	0
	Max	0	4	4	4	4
Placebo group	Mean	0.0	1.2	1.0	1.0	0.9
	Std	0.0	0.5	0.7	0.9	0.9
	*N*	105	105	105	105	105
	Min	0	0	0	0	0
	Max	0	2	4	4	4
*P* value		N/A	0.6905	0.1638	<0.0001	<0.0001

Baseline *P* value is from *t* test; non-baseline *P* values are LSMeans by visit; test = repeated measure ANOVA, main effect of treatment *P* < 0.0001; treatment by visit interaction *P* < 0.0001

*Min* minimum, *Max* maximum, *Std* standard deviation

**Table 10:** Flare score by treatment and visit (ITT)

		Baseline	Day 1	Day 3	Day 7	Day 14
Nepafenac group	Mean	0.0	0.9	0.5	0.3	0.1
	Std	0.0	0.6	0.6	0.5	0.3
	*N*	106	105	105	105	105
	Min	0	0	0	0	0
	Max	0	2	2	2	2
Placebo group	Mean	0.0	0.9	0.7	0.8	0.6
	Std	0.0	0.5	0.7	0.8	0.8
	*N*	105	105	105	105	105
	Min	0	0	0	0	0
	Max	0	2	3	3	3
*P* value		N/A	0.5793	0.0041	<0.0001	<0.0001

Baseline *P* value is from *t* test; non-baseline *P* values are LSMeans by visit; test = repeated measure ANOVA, main effect of treatment *P* < 0.0001; treatment by visit interaction *P* < 0.0001

*Min* minimum, *Max* maximum, *Std* standard deviation

**Table 11:** Cell count measured by LFCM by treatment and visit (ITT)

		Day 1	Day 3	Day 7	Day 14
Nepafenac group	Mean	39.2	19.8	9.7	6.0
	Std	79.2	25.1	11.2	9.8
	*N*	54	54	54	54
	Min	0.0	0.0	0.0	0.0
	Max	428.7	116.4	52.7	65.3
Placebo group	Mean	40.4	55.3	32.2	24.1
	Std	52.8	66.2	37.3	31.4
	*N*	53	54	54	54
	Min	0.0	0.0	0.0	0.0
	Max	312.2	311.2	177.3	128.0
*P* value		0.8738	<0.0001	0.0107	0.0394

Non-baseline *P* values are LSMeans by visit; test = repeated measure ANOVA, main effect of treatment *P* = 0.0011; treatment by visit interaction *P* = 0.0186; cell count was measured by laser flare cell meter at six sites

*Min* minimum, *Max* maximum, *Std* standard deviation

**Table 12:** Flare value measured by LFCM by treatment and visit (ITT)

		Day 1	Day 3	Day 7	Day 14
Nepafenac group	Mean	20.6	20.6	13.4	11.4
	Std	24.6	40.9	12.2	9.1
	*N*	101	103	103	103
	Min	0.5	0.3	0.4	0.3
	Max	153.2	396.7	57.8	57.8
Placebo group	Mean	24.3	47.5	65.6	52.0
	Std	34.4	49.8	77.7	69.9
	*N*	101	104	104	104
	Min	1.8	2.5	2.5	1.5
	Max	307.6	333.9	479.7	479.7
*P* value		0.6201	<0.0001	<0.0001	<0.0001

Non-baseline *P* values are LSMeans by visit; test = repeated measure ANOVA, main effect of treatment *P* < 0.0001; treatment by visit interaction *P* < 0.0001

*Min* minimum, *Max* maximum, *Std* standard deviation

The incidence of treatment failures 14 days after surgery was 1.9% (2/106) in the nepafenac group and 8.6% (9/105) in the placebo group. There were more subjects who had no response to treatment in the placebo group than in the nepafenac group, although no significant difference was observed between groups (Table 13).

**Table 13:** Incidence of treatment failures at each study visit (ITT)

Treatment	Total	Day 1		Day 3		Day 7		Day 14	
	*N*	*N*	%	*N*	%	*N*	%	*N*	%
Nepafenac group	106	2	1.9	2	1.9	2	1.9	2	1.9
Placebo group	105	0	0.0	1	1.0	9	8.6	9	8.6
*P* value			0.5467		0.3974		0.2001		0.2236

NLMixed model *P* = 0.6151; main effect of treatment; failure defined as aqueous cells score ≥3 or aqueous flare score = 3 or ocular pain score ≥4

### Safety

A total of 26 AEs were reported in 21 subjects (19.6%) in the nepafenac group and 31 AEs were reported in 24 subjects (22.4%) in the placebo group. Two AEs in the nepafenac group and six AEs in the placebo group were regarded as adverse reactions (ADRs) for which the causal relation could not be ruled out. The ADRs reported in the nepafenac group were foreign body sensation in the eyes in one subject (0.9%) and eye discharge in one subject (0.9%), both of which were mild and non-serious. The ADRs reported in the placebo group were eye pruritus in one subject (0.9%), foreign body sensation in the eyes in one subject (0.9%) subject, eye irritation in two subjects (1.9%), and increased lacrimation in two subjects (1.9%), all of which were non-serious. All of the ADRs were mild in severity, except for moderate foreign body sensation in the eyes reported in one subject. In the nepafenac group, no clinically relevant findings were obtained in other examinations for safety evaluations including laboratory tests, blood pressure/pulse rate, BCVA, IOP, slit lamp examination, and fundus examination.

## Discussion

In Japan, the ophthalmic preparations of steroids or NSAIDs are used for the treatment of postoperative inflammation following cataract surgery. NSAIDs are also used for the prevention of postoperative cystoid macular edema (CME). Diclofenac sodium and bromfenac have been shown to reduce postoperative inflammation [7–10]. Some studies have shown that aqueous flare values in the early postoperative period are even lower in those treated with diclofenac sodium than in those treated with steroids [11, 12]. Other studies have demonstrated that NSAIDs are effective in preventing postoperative CME [13–16], in which an ophthalmic NSAID was administered for several months after cataract surgery. Nepafenac is a prodrug metabolized into its active form, amfenac. Ex vivo inhibition of prostaglandin synthesis by nepafenac/amfenac was significantly greater and of longer duration than diclofenac [2]. And nepafenac had sixfold greater corneal penetration than diclofenac [17]. Amfenac was the more potent COX-2 inhibitor than bromfenac and ketorolac. And the combined area under the curve of nepafenac and amfenac was higher than the bromfenac and ketorolac [1].

In this study, nepafenac expected to prevent postoperative inflammation and ocular pain, was administered as a monotherapy to patients undergoing cataract surgery, and its efficacy and safety in the management of postoperative inflammation and ocular pain were compared with those of a placebo. The subjects were Japanese patients with cataracts who were undergoing cataract surgery with PEA and IOL implantation. Evaluations were based on cure rates 14 days after surgery and ocular pain-free rates during the first 14 days after surgery. The cure rate was 71.4% in the nepafenac group and 28.6% in the placebo group, showing a significant difference between groups. Those who achieved cures in the placebo group appeared to have undergone spontaneous resolution of postoperative inflammation, as indicated by a gradual increase in cure rates over the course of treatment. In contrast, the nepafenac group demonstrated higher cure rates than those in the placebo group, with significant intergroup differences observed on days 7 and 14 postoperatively. This study revealed significant differences in cure rates between nepafenac and placebo over the course of treatment, which was consistent with the results of a study conducted in the USA using the same evaluation [4].

The use of LFCM in the evaluation of postoperative inflammation following cataract surgery has been described in many studies [18–20]. LFCM enables assessment of the degree of inflammation by quantitatively measuring such parameters as white blood cell count and protein concentration in the aqueous humor. This study also included secondary endpoints that were evaluated with LFCM. Aqueous cell counts and aqueous flare values as measured with LFCM were significantly lower in the nepafenac than in the placebo group. The mean flare value in the placebo group reached its peak on day 7 while no postoperative increase in flare values was observed in the nepafenac group. Cell scores and flare scores determined by slit lamp biomicroscopy and flare values and cell counts measured with LFCM showed similar patterns of changes over time from day 3 and thereafter, demonstrating the appropriateness of the efficacy evaluation based on cell scores and flare scores determined by slit lamp biomicroscopy and the efficacy of nepafenac in the management of postoperative inflammation.

Since postoperative inflammation following intraocular surgery contributes to the development of CME, it is very important to reduce or resolve postoperative inflammation. In this study, evaluations were only performed up to 2 weeks after surgery, and thus the preventative effect of nepafenac on CME was not examined. However, the fact that the cure rate of nepafenac was significantly higher than placebo implies that nepafenac is likely to be effective in preventing the development of CME.

This study also examined the ocular pain after cataract surgery. The pain-free rate was 96.2% in the nepafenac group and 67.6% in the placebo group, showing a significant difference between groups. Most subjects in the nepafenac group complained of no ocular pain throughout the study period while more than 30% of the subjects in the placebo group complained of ocular pain. These findings also suggest the need for the postoperative use of agents with an analgesic effect, such as nepafenac 0.1%, in clinical practice.

Reported AEs associated with ophthalmic NSAIDs include diffuse superficial keratitis, corneal erosion, and corneal epithelium disorder, and more serious AEs include corneal ulcer and corneal perforation [21]. In this study, no clinically significant AEs associated with nepafenac were reported, and the incidences of AEs and ADRs in the nepafenac group were lower than those in the placebo group, demonstrating the favorable safety profile of nepafenac.

## Conclusion

Nepafenac ophthalmic suspension 0.1% is significantly more effective than the placebo in the management of postoperative inflammation and ocular pain following cataract surgery and thus is considered an essential NSAID in ophthalmic surgery.
